# MRI-Guided Online Adaptive Stereotactic Body Radiation Therapy of Liver and Pancreas Tumors on an MR-Linac System

**DOI:** 10.3390/cancers14030716

**Published:** 2022-01-30

**Authors:** Teo Stanescu, Andrea Shessel, Cathy Carpino-Rocca, Edward Taylor, Oleksii Semeniuk, Winnie Li, Aisling Barry, Jelena Lukovic, Laura Dawson, Ali Hosni

**Affiliations:** 1 Princess Margaret Cancer Centre, University Health Network, Toronto, ON M5G 2M9, Canada; andrea.shessel@rmp.uhn.ca (A.S.); cathy.carpino-rocca@rmp.uhn.ca (C.C.-R.); edward.taylor@rmpuhn.ca (E.T.); oleksii.semeniuk@rmp.uhn.ca (O.S.); winnie.li@rmp.uhn.ca (W.L.); aisling.barry@rmp.uhn.ca (A.B.); jelena.lukovic@rmp.uhn.ca (J.L.); laura.dawson@rmp.uhn.ca (L.D.); ali.hosni.abdalaty@rmp.uhn.ca (A.H.); 2 Department of Radiation Oncology, University of Toronto, Toronto, ON M5T 1P5, Canada; 3 Department of Mechanical and Industrial Engineering, University of Toronto, Toronto, ON M5S 3G8, Canada

**Keywords:** MRI, MR-guided radiation therapy, MR-Linac, liver metastases, HCC, pancreas, SBRT, MRIgRT, MRgRT, susceptibility, numerical simulations, MR image distortions

## Abstract

**Simple Summary:**

The hybrid magnetic resonance imaging and medical linear accelerator (MR-Linac) systems are expected to revolutionize radiation therapy, uniquely offering high quality soft-tissue contrast and fast imaging to facilitate the online re-planning and guidance of the treatment delivery. While the clinical procedures for stereotactic body radiotherapy are well-established for conventional linacs (with their strengths and weaknesses), they still require significant development and refinement for the MR-Linac systems. Adjustment of fractionation schemes including clinical goals, patient selection, organ motion management, treatment session length, staff logistics, and overall complexity of the online re-planning sessions are key factors that drive the adoption of MR-Linac technologies. In this report, we present the clinical implementation of an MRI-guided radiation therapy workflow, which was used to treat 16 upper gastro-intestinal cancer patients on a 1.5 T MR-Linac platform. The workflow was proven to be feasible for a wide range of clinical scenarios, and the overall treatment session time was significantly reduced as tasks were optimized and the clinical team gradually gained expertise.

**Abstract:**

Purpose: To describe a comprehensive workflow for MRI-guided online adaptive stereotactic body radiation therapy (SBRT) specific to upper gastrointestinal cancer patients with abdominal compression on a 1.5T MR-Linac system. Additionally, we discuss the workflow’s clinical feasibility and early experience in the case of 16 liver and pancreas patients. Methods: Eleven patients with liver cancer and five patients with pancreas cancer were treated with online adaptive MRI-guidance under abdominal compression. Two liver patients received single-fraction treatments; the remainder plus all pancreas cancer patients received five fractions. A total of 65 treatment sessions were investigated to provide analytics relevant to the online adaptive processes. The quantification of target and organ motion as well as definition and validation of internal target volume (ITV) margins were performed via multi-contrast imaging provided by three different 2D cine sequences. The plan generation was driven by full re-optimization strategies and using T2-weighted 3D image series acquired by means of a respiratory-triggered exhale phase or a time-averaged imaging protocol. As a pre-requisite for the clinical development of the procedure, the image quality was thoroughly investigated via phantom measurements and numerical simulations specific to upper abdominal sites. The delivery of the online adaptive treatments was facilitated by real-time monitoring with 2D cine imaging. Results: Liver 1-fraction and 5-fraction online adaptive session time were on average 80 and 67.5 min, respectively. The total session length varied between 70–90 min for a single fraction and 55–90 min for five fractions. The pancreas sessions were 54–85 min long with an average session time of 68.2 min. Target visualization on the 2D cine image data varied per patient, with at least one of the 2D cine sequences providing sufficient contrast to confidently identify its location and confirm reproducibility of ITV margins. The mean/range of absolute and relative dose values for all treatment sessions evaluated with ArcCheck were 90.6/80.9–96.1% and 99/95.4–100%, respectively. Conclusion: MR-guidance is feasible for liver and pancreas tumors when abdominal compression is used to reduce organ motion, improve imaging quality, and achieve a robust intra- and inter-fraction patient setup. However, the treatment length is significantly longer than for the conventional linac, and patient compliance is paramount for the successful completion of the treatment. Opportunities for reducing the online adaptive session time should be explored. As the next steps, dose-of-the-day and dose accumulation analysis and tools are needed to enhance the workflow and to help further refine the online re-planning processes.

## 1. Introduction

Commercially available MR-Linac systems integrating a low [1] or high [2] field magnetic resonance imaging (MR or MRI) scanner with a radiation therapy linear accelerator (Linac) on a common supporting gantry have been gaining wider adoption due to their ability to use MR image data to design and execute online adaptive treatment sessions. Additional research prototypes explore various system configurations and their clinical relevance [3,4]. MR-Linacs represent a radical progression from the conventional Linac platform operating with on-board X-ray-based imagers relying on cone-beam computed tomography (CBCT) methods. The advantage of MR-Linac technologies is expected to be not only in the MRI’s superior image quality (spatial and temporal) as compared to CBCT but also in their procedural flexibility for performing online re-planning using data acquired on-demand and real-time guidance of the treatment delivery.

Specific to upper gastro-intestinal (UGI) cancer sites, there are multiple stringent requirements influencing the quality of the radiation therapy (RT) treatment design and delivery: (a) soft-tissue contrast to visualize and accurately delineate the cancerous targets concomitant with adjacent organs-at-risks (OAR), (b) quantification of organ motion across a wide range of scenarios (magnitudes of mm to cm), (c) motion management and robust reproducibility of patient setup, (d) tracking and constant monitoring of intra- and inter-fractional anatomical changes, and (e) the ability to promptly intervene to adjust or adapt the treatment based on real-time feedback. Imaging is the de facto method for providing the anatomical and functional information required by the RT workflow, and MRI is the best candidate to fulfill this role. Preliminary work stressed the benefits of MR guidance [5,6].

Previous studies investigated the feasibility of MRI-guided online adaptive treatment workflows for the Unity 1.5T MR-Linac system (Elekta, Stockholm, Sweden) as applied to UGI cancer sites. Liver and pancreas patients were treated in free-breathing using motion-averaged or mid-position 3D image data derived from retrospectively reconstructed 4D MRI data [7]. Pancreas patients with abdominal compression were treated using a single prescription of 50 Gy in five fractions and motion-averaged 3D image data [8].

In the present study, we present a new and comprehensive workflow implementation for the Unity MR-Linac system as well as our early experience with treating 16 liver and pancreas cancer patients. Patient setup was established with abdominal compression and multiple prescription regiments were available for treatment plan design. The MRI-guided online adaptive sessions were facilitated by (a) fast imaging acquired with three different 2D cine sequences providing different image contrasts to visualize the target and quantify treatment margins and (b) 3D planning data acquired in either the exhale phase of the respiratory cycle or time-averaged depending on each patient’s characteristics.

## 2. Materials and Methods

Eleven liver and five pancreas cancer patients were treated using stereotactic body radiation therapy (SBRT)-based online adaptive procedures on a Unity 1.5T MR-Linac system. The SBRT prescriptions to pancreatic cancer were based on five fractions while the liver cases were planned with either single or five fraction regiments. The detailed patient specifics are provided in Table 1. The patient image data were collected under a study approved by the research ethics board at our institution.

### 2.1. Quantification of Image Spatial Accuracy

The development of the UGI clinical protocol for the Unity MR-Linac system at our institution relied on (a) the assessment of the imaging system and optimization of imaging sequences specific to liver and pancreas sites and (b) the integration of imaging with treatment planning, organ motion monitoring, and online guidance for treatment delivery. The spatial accuracy of the Unity-MR images was quantified via phantom measurements and numerical simulations. The composite image distortions due to system-related and patient-induced distortion fields were assessed for all clinical scenarios: 2D cine for fast sampling of organ and target motion, 3D acquisitions for planning, and simulated 4D data for facilitating the accurate generation of internal target volume (ITV) margins. Specifically, the MRID^3D^ phantom (Modus QA, London, UK, ON), which is based on harmonic analysis [9], was used to quantify system-related distortion fields due to gradient non-linearities (GNL) and magnetic field uniformity (i.e., *B*_0_ mapping) in a large imaging field of view (FOV) [10]. Patient-induced geometric distortions caused by variation in tissue magnetic susceptibility (*χ*) properties were modeled numerically by means of finite difference methods (FDM), using patient-specific susceptibility maps derived from CT images [11]. The numerical computations were performed on 3D data sets corresponding to the extreme exhale and inhale phases of the respiratory cycle to characterize the maximum extent of the *χ* effects present during real-time organ motion. The 4D composite distortion fields, combining both the scanner and patient related sources of image artifacts, were assessed for a typical liver case [12]. The geometric distortions were evaluated in terms of mean and maximum (max) values.

### 2.2. Assessment of Imaging for the Quantification of Organ Motion

The imaging specifications relevant to the quantification of organ motion were investigated using the Zeus 008Z phantom (CIRS, Norfolk, VI, USA), which provides anthropomorphic internal structures with known geometry. The phantom also allows for 4D motion (i.e., translation and rotation) via an ensemble consisting of an actuator, a connecting piston, and a cylindrical container with contrast material and a surrogate target. The phantom was programmed with pre-defined motion traces for scanning and the traces were subsequently applied as baselines for assessing the performance of MR imaging.

2D cine MR imaging was initially evaluated as it is a fast and straightforward tool to probe organ motion. The Unity treatment session manager (TSM) relies only on one 2D cine sequence for real-time motion monitoring during the online adaptive session, i.e., balanced transfer fast field echo (btFFE). The key parameters of this sequence were TE/TR of 1.7/3.4 ms, flip angle of 40°, in-plane resolution of 3 × 3 mm^2^, and slice thickness of 5 mm. Since the btFFE sequence was found to be insufficient for the UGI patient volunteers investigated in the early phase of the clinical protocol development, two other 2D cine sequences based on T1w and T2w contrast were developed and optimized in-house. The T1w-cine sequence had TE/TR of 2.3/4.7 ms, a flip angle of 25°, and in-plane pixel resolution of 2.5 × 2.5 mm^2^, and 8 mm for the slice thickness. The T2w-cine sequence specifications were TE/TR of 80/500 ms, a flip angle of 90°, and in-plane pixel resolution of 2.5 × 2.5 mm^2^, and slice thickness of 8 mm^3^. The main 3D image data sets required for RT planning were acquired with two main sequences: (a) T2w navigator triggered sequence (T2w-Nav) with data collected in the exhale phase-TE/TR of 247/2100 ms, flip angle of 90°, and voxel resolution of 2 × 2 × 2 mm^3^ and (b) T2w time-averaged sequence (T2w-Ave), which reconstructs a blurred scan-TE/TR of 104/1300 ms, flip angle of 90° and voxel resolution of 1.2 × 1.2 × 2 mm^3^. All 2D cine and 3D sequences were first tested using the Zeus 4D motion phantom to assess the representation of motion in the reconstructed image series. The sequences were further tested in healthy and patient volunteers to determine their value for rendering OARs and targets as well as for establishing guidelines for their clinical use relative to magnitude of organ motion present in the RT setup. The 4D motion phantom was also used to perform the end-to-end testing for all clinical workflow development work.

### 2.3. Imaging for Patient Simulation

All patients underwent three imaging sessions consisting of Unity-MR, 3T MR Skyra-Fit (Siemens, Erlangen, Germany), and Brilliance Big Bore CT scanner (Philips Medical Systems, Cleveland, OH, USA). The patient setup was with arms down and under abdominal compression (AC) using an MR-compatible pressure belt and manual pump system (Orfit, Wijnegem, Belgium). The Unity-MR was the first imaging session in the workflow as it was key for triaging patients for MR-guided online adaptive sessions versus CBCT-based image guided radiation therapy (IGRT) on a conventional linac. The criteria for Unity were based on (a) motion management and patient compliance with AC and (b) quality of imaging. Patients who were suitable for the active breathing control (ABC) technique were routinely treated on the conventional linac since the ABC device was not MR safe and overall treatment margins were minimal. However, carefully selected ABC-feasible patients were chosen for the MRL-based treatment when (a) motion under AC was small and (b) the target was in close proximity to dose-limiting luminal structures and the ABC technique was deemed not to improve the safety of SBRT delivery—e.g., cases with expected significant inter-fractional spatial changes, which would degrade the quality of conventional IGRT.

The Unity-MR scanning session included fast imaging using 2D cine sequences for the assessment of residual organ motion under AC. The maximum allowed amplitude was 1.5 cm. The fast imaging relied on the btFFE, T1w-cine, and T2w-cine sequences (see Section 2.2), which were acquired in the sagittal and/or coronal planes. Of note, the largest dimension of the cine image voxel (i.e., 5–8 mm) was not in the dominant direction of the superior–inferior direction. 3D imaging was also performed as required for the reference plan using both the T2w-Nav and T2w-Ave sequences. The most optimal 3D data set drove the design of the online adaptive treatment. Additional sequences such as T2w 2D multi-slice, T1w navigator-triggered, and DWI/IVIM were also acquired to support research activities.

The 3T MR session consisted of 2D cine, 3D acquisitions with and without contrast, as well as multi-phase imaging such as arterial, venous, and venous 3-min delayed. The CT session included 4D CT in addition to multi-phase imaging similar to 3T MR.

### 2.4. Reference Planning

All simulation data was imported first into the RayStation (RaySearch, Stockholm, Sweden) treatment planning system (TPS) for pre-processing and generation of reference contours. This allowed for a more efficient management of multiple multi-modality data sets (at least 12 image series) via a Python-based scripting interface. The availability of data in RayStation also facilitated the rapid re-planning of cases for the conventional linac in case of prolonged downtime of the Unity system as RayStation is the main treatment planning system at our institution outside the Unity environment. The internal target volume (ITV) margins were defined using the Unity-MR cine scans and 4D CT data. The planning target volume (PTV) was defined as a 5 mm uniform geometric expansion of either GTV (T2w-Ave) or ITV (T2w-Nav) in all directions.

The planning MR and CT image series along with a full set of contours were subsequently transferred to Monaco v5.4 (Unity’s TPS) for the design and generation of the reference plan. The exhale phase of the 4D CT was selected as the primary data set for only the first three cases. As the team gained more experience, the reference planning was established with MR as the primary image set. For this, CT data was used solely to derive and assign bulk electron density information to three anatomical structures, which were relevant for dose computations—i.e., external patient contour, liver, and lungs. The same information was propagated forward to the daily adaptive sessions using online data. The plans were generated using 8–12 IMRT fields and full inverse optimization using a 3 mm dose grid size and 1% statistical uncertainty for the Monte-Carlo-based dose calculation. Templates for each fractionation regime were applied as per clinical protocols. In this study, the liver patients were treated using multiple dose levels between 27.50 Gy and 45 Gy in 5 fractions or 16 Gy and 24 Gy in single fractions. Of note, 30 Gy in 1 fraction was also available, and one patient was treated with this regiment; however, the data was not included in the present work as the treatment was interrupted by a machine breakdown. An additional treatment session had to be scheduled at a later time with a completion plan reflecting the remainder of the planned dose. The pancreas cancer patients were treated with either 30 Gy or 40 Gy in five fractions with the possibility of a simultaneous integrated higher dose to hypoxic sub-volumes defined by FAZA-PET MRI [13]. The prescription specifics per case are shown in Table 1.

### 2.5. Online Adaptive Workflow

**Figure 1:**
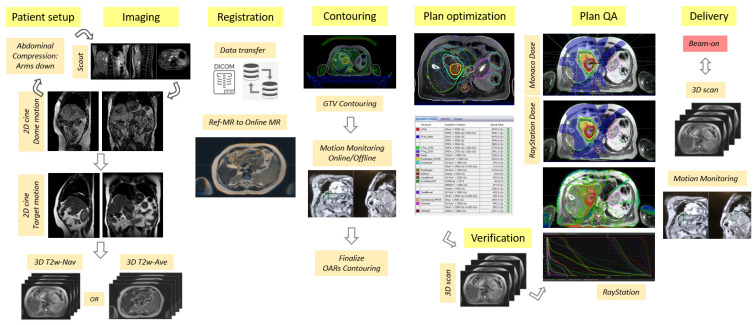
Flow chart highlighting the key processes in the MRI-guided online adaptive workflow based on the adapt-to-shape (ATS) procedure for UGI sites on the Unity MR-Linac system.

The flow diagram for the online adaptive workflow is shown in Figure 1. The RT team consisted of two MR-RT cross-trained radiation therapists, one medical physicist, and one radiation oncologist. The patient setup per each treatment session was established by matching the belt and pump pressure values documented at the simulation stage. The setup was further verified via 2D cine imaging performed in the sagittal and coronal planes on the liver dome. 2D cine was subsequently performed on the target to verify ITV margins. The 2D cine sequence for the quantification of target motion was identified at the simulation stage to best visualize the target and surrounding anatomical landscape. Once data was brought into Online Monaco, the reference and daily MR data were co-registered, and contours were transferred via either rigid or deformable registration. Except for the full liver organ, the contouring of all OARs was performed within the ContourAid region of interest (ROI), defined as a 2–4 cm uniform expansion of the PTV. This was acceptable as the clinical goals were based on maximum dose values. An additional ROI labeled as Tracking was propagated from the reference plan to the online plan to represent the allowed target motion within the PTV boundaries. This ROI was displayed by the Unity’s motion monitoring environment via 2D cine imaging in all three central axis planes and used to monitor the patient setup during the treatment session. The planning was based on Unity’s adapt-to-shape (ATS) strategy, which involved the full re-optimization of a new plan using pre-defined core specifications such as the beam configuration, prescription, optimization parameters, and clinical goals. The physics plan QC and secondary monitor unit (MU) calculations were performed in RayStation by means of a semi-automated process based on scripting. The plan approval by all members of the clinical team was implemented via a web-based application. Additional 3D data were collected for plan verification while the plan was transferred to Mosaiq (Elekta, Stockholm, Sweden), i.e., a record and verify system, and prepared for delivery. A verification image was acquired before start of treatment, with the PTV rigidly fused to the new data set to assess coverage. Motion monitoring was engaged several times during the online process to confirm setup stability. This process was repeated during the plan delivery. 3D data were collected one more time during beam-on to support the development of future dose-of-the-day (DOTD) and dose accumulation processes. Patient-specific QA (PSQA) measurements were performed for the reference plan and post each treatment fraction using ArcCheck MR (Sun Nuclear, Melbourne, FL, USA). The results were reviewed considering the absolute dose (AD) and relative dose (RD) metrics prior to the start of next session.

## 3. Results

**Figure 2:**
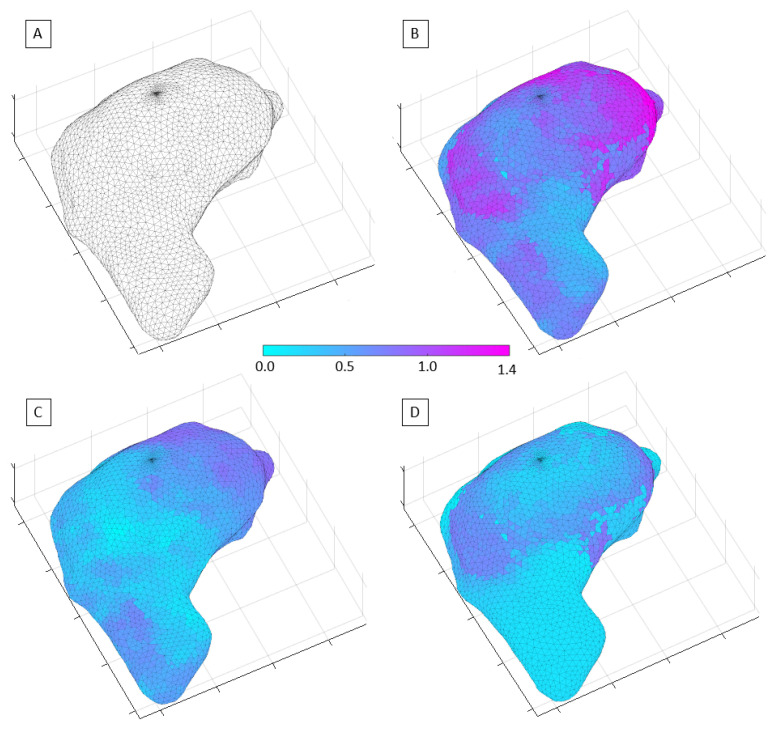
Representation of the MR image distortion fields relevant to liver RT and imaging performed on the Unity-MR system: (**A**) 3D mesh of the liver volume used to plot the distortion field distribution; (**B**) total distortion values; (**C**) system-related spatial distortions; (**D**) susceptibility-induced geometric distortions.

Figure 2 shows the exhale phase of a liver case, which was used to assess the image quality expected in the Unity online adaptive RT workflow. The mean and max system-related distortions due to GNL and *B*_0_ were found to be within 0.3 mm and 1.1 mm, respectively. The susceptibility-induced distortions were found to be 0.2/1.2 mm for mean/max. Given that the *χ* values apply only along one axis (typically x- or y-axis), the composite total distortions amounted to approximately mean/max of 0.4/1.4 mm. The modeling for the inhale phase of the liver showed negligible variations when compared to the exhale phase.

**Figure 3:**
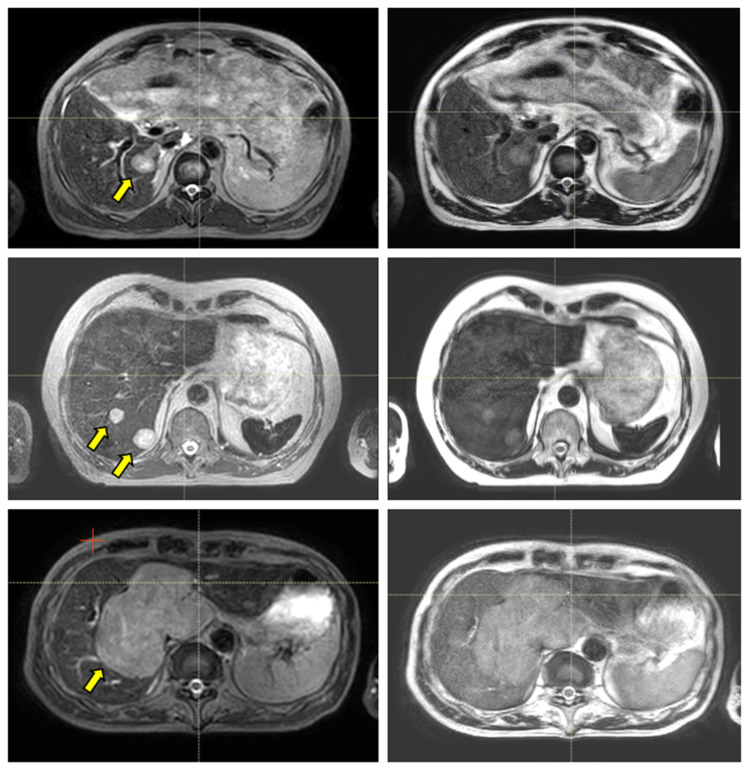
Liver examples depicting the comparison between the T2w-Nav (**first column**) and T2w-Ave (**second column**) image sequences. The target(s) location is identified by the yellow arrow. The samples correspond to Patients 7, 8, and 9 as per Table 1. These cases were planned with the T2w-Nav image data.

**Figure 4:**
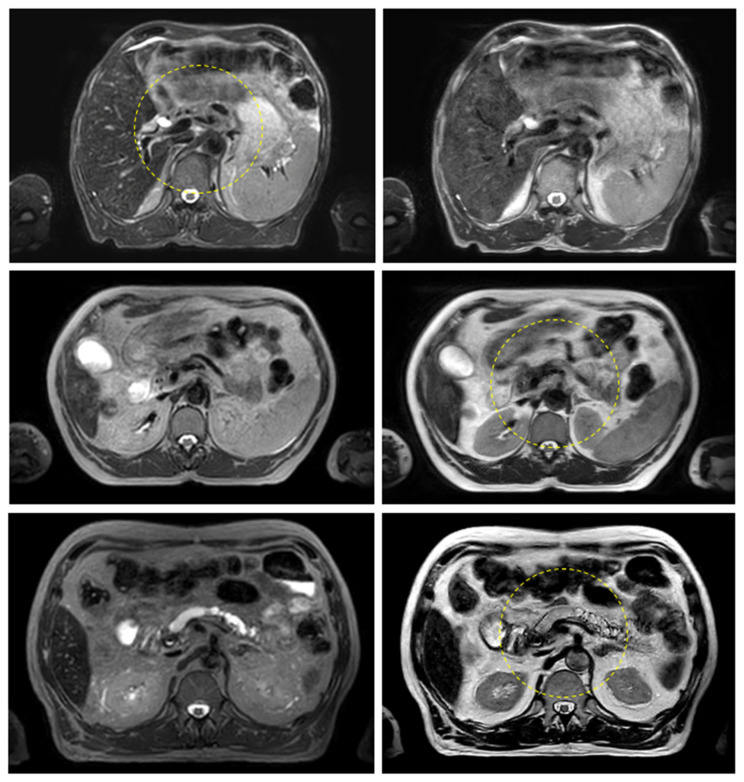
Pancreas examples highlighting the comparison between the T2w-Nav (**first column**) and T2w-Ave (**second column**) image sequences. The target location is identified by the yellow dashed circle. The planning data set was the T2w-Nav for the first case and T2w-Ave for the second and third case. The samples correspond to Patients 11, 14, and 16 as listed in Table 1.

Figure 3 and Figure 4 show the comparison between the T2w-Nav and T2w-Ave sequences acquired during the Unity-MR simulation sessions for several liver and pancreas cases, respectively. T2w-Nav required on average 5–6 min to acquire a full image series depending on each patient’s breathing pattern. In contrast, the T2w-Ave acquisition time was fixed to roughly 4 min. In certain instances, the T2w-Ave scan time was increased by 1–2 min to provide extended superior–inferior coverage. In particular, Figure 3a shows the two liver targets bundled together while Figure 3b depicts two out of four targets present in the same imaging plane. The post-operative targeted areas with RT for three pancreas cases are shown in Figure 4.

**Figure 5:**
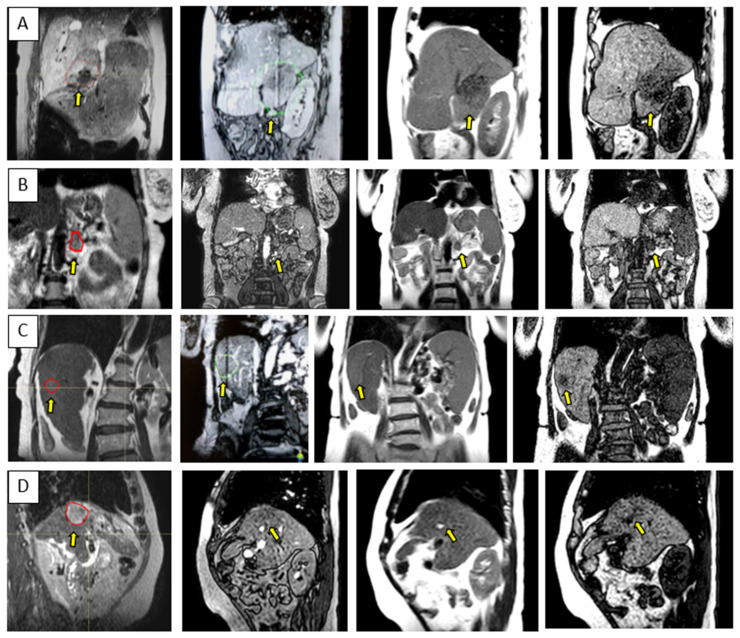
Sample data for four UGI patients highlighting the range of contrast available with the 3D cine sequences used for the quantification and monitoring of patient setup and target motion. Each row is for a different patient and shows either a coronal or sagittal view: (**A**–**D**) corresponds to Patients 10, 15, 6, and 12, as per Table 1. The columns correspond from left to right to the MR planning data set identifying the target location, btFFE cine, T2w-cine, and T1w-cine.

The ability to visualize moving targets with 2D cine imaging is depicted in Figure 5 for one pancreas case and three liver cases, as acquired with the btFFE, T1w-cine, and T2w-cine sequences. The quality of the cine data was critical as this imaging was used to derive and verify ITV margins and monitor setup during the online treatment sessions.

**Figure 6:**
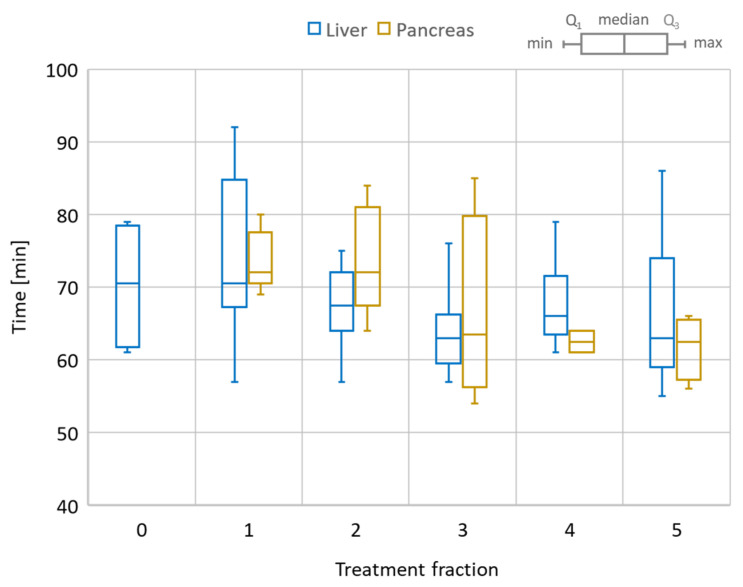
Total session time for the online adaptive workflow. Comparison between liver and pancreas cases. Liver also includes the dry-run (Day 0) session time as a reference. The box plot shows the min/max/median values as well as the first (Q_1_) and third (Q_3_) quartile of the session time distribution.

The total session time for the online adaptive session is shown in Figure 6 for all liver and pancreas treatments. The median/range values for liver 1-fraction and 5-fraction regiments were 80/70–90 min and 67.5/55–90 min, respectively. For the pancreas, the mean/range values were 68.2/54–85 min. Of note, the first three liver cases had the Day 0 session as well, for which the mean/range recorded values were 70.3/61–68 min.

**Figure 7:**
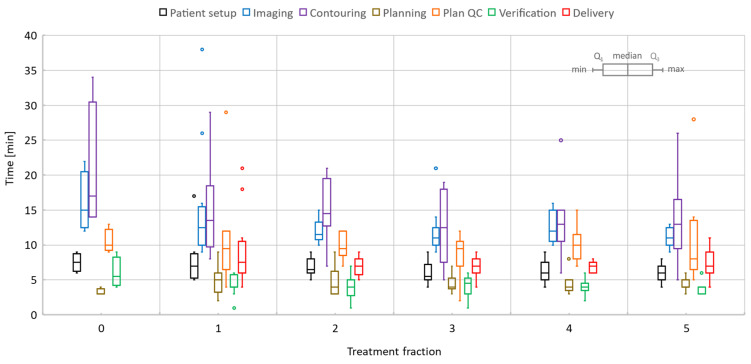
Liver online adaptive sessions for Fractions 1 and 5 and including the dry-run (or Day 0) session where available. The time required to perform key processes in the adapt-to-shape workflow.

**Figure 8:**
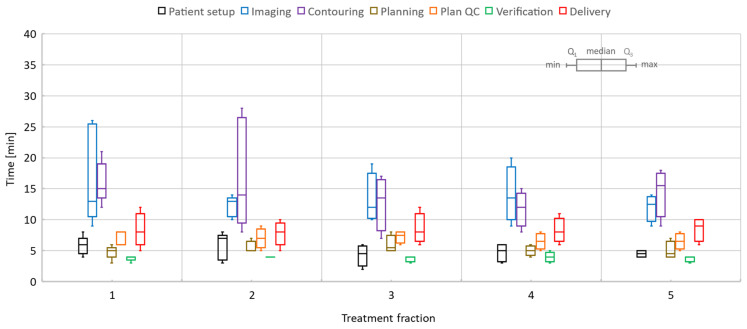
Pancreas online adaptive sessions, all including five fractions. The time spent on key processes in the adapt-to-shape workflow.

Figure 7 and Figure 8 show detailed data for key online processes corresponding to liver and pancreas given the specific scenarios summarized in Table 1. Considering all fractions and prescriptions for liver, the mean/range patient setup time was 6.7/4–17 min, imaging time was 12.5/9–26 min, image registration (rigid/deformable) and contouring required 14.4/5–34 min, plan optimization time was 4.6/2–9 min, physics plan QA time was 10/2–29 min, verification time was 4.2/1–9 min, and treatment delivery time was 7.5/4–21 min. The imaging time included multiple steps: scout, 2D cine on dome and target, measurements to quantify the motion in the setup with abdominal compression and target, and 3D image acquisition for planning. Notably, the minimum and maximum delivery time corresponded to 27.5Gy/5-fractions and 24Gy/1-fraction treatments, respectively.

Similarly, for pancreas, the patient setup time was 5.1/2–8 min, imaging took 13.8/9–26 min, registration and contouring time was 14.6/7–28 min, planning time was 5.3/3–8 min, physics plan QA time was 6.9/5–9 min, verification time was 3.9/3–5 min, and treatment delivery time was 8.3/5–12 min. When the T2w-Ave scan was selected as the planning data set, an additional 3D scan with T1-weighted contrast (2 min long) was acquired to activate the online TSM workflow since the T2w-Ave sequence was not part of the pre-defined TSM exam cards. Plan QA and secondary MU calculations included issues related to data transfer and post-processing in RayStation. The treatment time also included beam faults and associated tasks required to remediate and resume the beam delivery.

The overall time per process stayed roughly the same over the five-fraction course of treatments for both liver and pancreas. Since there was a certain degree of parallel execution of tasks, the varying timing for starting and completing the tasks led to an overall decrease in the total length of the treatment time for most of the liver and pancreas cases. 

The PSQA results are summarized in terms of mean AD and RD values per patient treatment in Table 1. The mean/range of AD and RD values for all treatment sessions were 90.6/80.9–96.1% and 99/95.4–100%, respectively.

**Figure 9:**
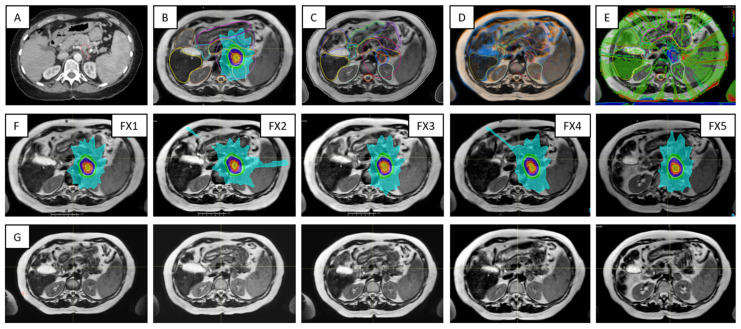
Highlights of the online adaptive workflow for a pancreas case —i.e., Patient 10 in Table 1: (**A**) CT image required for the assignation of bulk electron density values to contours on MR image data; (**B**) MR-based reference plan; (**C**) contours defined during the first treatment fraction; (**D**) image registration between MR images acquired for planning and verification for Fraction 1; (**E**) dose difference map representative for the secondary MU calculations; (**F**) the row shows the ATS plan for each of the five treatment fractions; (**G**) verification images corresponding to data from (**F**).

Figure 9 outlines key processes and various types of data required for the Unity treatments in the case of a pancreas patient. For each ATS data set acquired for planning, there was at least one verification scan acquired prior the treatment delivery and one other scan collected during beam on. The first verification scan was used to confirm stability of the patient setup and adequate representation of the ATS plan at the time of delivery, while the beam on time scan was used for research activities related to dose-of-the-day and dose accumulation.

## 4. Discussion

The distribution of the spatial distortions varies depending on its source. The system-related distortions increase slowly with distance from the MR isocenter, and, therefore, for liver, located well off-center, they manifest primarily as a scalar systematic shift across most of the liver volume. The *χ*-induced effects arise in the proximity of the diaphragm since they are caused by significant changes in tissue’s magnetic properties between lung and liver. Therefore, *χ* distortions are highly localized, and their distribution varies slightly with the shape of the interface surface and its relative orientation with respect to *B*_0_ [11]. Overall, the composite image distortions were considered to have a negligible impact on the planning activities for both liver and pancreas cases. The distortions were also negligible for all 2D cine imaging [12].

The quality of the target and OARs visualization varied per patient depending on motion characteristics. Figure 3 highlights the advantage of using T2w-Nav over the T2w-Ave for the liver cases. The T2-Nav performed best when motion was more than 5–6 mm. The signal collection was efficient and robust even when patient breathing under compression exhibited an irregular pattern. However, T2-Nav acquisition became impractical when there was little breathing motion as the triggering algorithm was not able to efficiently collect data in the exhale phase (long scanning time). Due to a slightly different T2w contrast and robust data acquisition under low organ motion amplitude, the T2-Ave scan was used as the main imaging method for pancreas. This is highlighted in Figure 4. T2-Ave became less efficient when large organ excursions were present as they introduced significant amounts of blur, which prevented the definition of both the target and OARs.

As depicted in Figure 5, the btFFE sequence often did not provide sufficient contrast to identify the target. Since this sequence is the only fast imaging option available for real-time monitoring in the Unity-TSM environment, any other cine sequence had to be acquired and evaluated on the MR console only. Thus, the target motion assessed with the T1w- and T2w-cine sequences was performed as an on-demand auxiliary process to the TSM. Another distinguishing factor was that the frame rates for T1w- and T2w-cine were 2 Hz and 3–4 Hz, respectively, compared to 5–6 Hz for btFFE. However, lengthening the cine acquisition up to 1 min allowed for the full sampling of the organ/target residual motion cycle under AC.

The patient setup with abdominal compression provided two main benefits to the online adaptive workflow. First, it decreased the target and organ motion, leading to improved imaging, and provided a robust setup over the full length of the treatment. Second, the intra- and inter-fraction motion matched well with initial values from the reference plans, with variations being within the spatial resolution of the cine imaging. Out of 75 total online adaptive sessions, the setup had to be adjusted only twice (different patients), as the dome motion was found to be larger than expected at the initiation of the workflow. The issue was corrected by adjusting the AC belt and its pressure. No systematic shifts in the targeted treatment volume were observed under AC. Of note, two patients who initially were good candidates for Unity were not able to tolerate the prolonged Unity-MR simulation sessions and had to be treated on the conventional linac with CBCT-based image guidance.

The overall handling of each five-fraction case improved gradually with each fraction and consolidation of prior knowledge regarding patient setup and patient’s compliance during the rather long online sessions, quality of imaging and target/OAR definition, and familiarity of the team with the case. For liver, the first three cases had a dry-run (Day 0) session for which processes such as imaging and quantification of motion on 2D cine, contouring, plan QA, and verification took significantly longer than for the subsequent online treatment sessions. This was expected as the clinical team was constantly refining tasks and building expertise. Similarly, the rather constant execution time for each process over five fractions was partly due to rotation of team staff (except for the radiation oncologist), who were not fully familiar with the case and required extra time to review online information.

The first three cases were planned with CT as the reference data set. Moving to planning directly on MR significantly helped the contour propagation from reference MR to online MR. Contour mapping via rigid and/or deformable registration worked well for the whole liver and had some benefits for target delineation. Most of luminal structures had to be manually adjusted or fully recontoured. Decreasing the spatial envelope of the contouring region to 2–4 cm expansion from PTV helped reduce the total contouring time.

In particular for liver, the contouring for single fraction cases went faster than for five-fraction sessions as the procedure targeted solitary tumors located within the liver mass and at least 1.5 cm away from any luminal structure. However, the single fraction beam delivery time increased at least 2–3-fold due to the flat dose rate. The beam delivery time in test cases went up to 18–21 min for the 30 Gy in one fraction regiment.

All treatment sessions were based on the ATS approach; however, the clinical workflow had the option to generate an adapt-to-position (ATP) plan in the eventuality that a small systematic shift occurred. The workflow also allowed for inter-fraction modifications of the ITV margins based on organ motion measurements with cine imaging. As additional flexibility in the workflow, any ATS plan could be used as a reference plan for subsequent sessions. This process was employed in the case of one patient when the quality of the contours was superior at the first fraction compared to the reference plan, and it was estimated to speed up the contour propagation for the remainder of the treatment. As a future direction, dose-of-the-day is currently being investigate as a potential clinical process aimed to enhance the current online procedure and facilitate the development of dose accumulation processes.

## 5. Conclusions

In this study, we discuss a comprehensive workflow for the treatment of UGI cancer patients with abdominal compression on the Unity 1.5T MR-Linac system. Following the investigation of composite image distortions and their effect on the OAR and target contouring, we suggest 2D cine and 3D image sequences to visualize the tumor sites and manage various degrees of organ motion. These findings are directly relevant to liver and pancreas cancer patients, while the overall approach to selecting the optimal image acquisition can be adapted to any other treatment site. Further, we provide insight into various online adaptive processes and total treatment session time for a wide range of fractionation regiments. Several recommendations for process improvement are provided as well as directions for future developments including dose-of-the-day and dose accumulation activities.

## Figures and Tables

**Table 1:** Summary of patient information and specifics regarding the online adaptive treatment session.

No	Age (Years)	Sex	Tumor Site	Prescription (Gy/Fractions)	Planning Sequence	SI Motion (mm)	PSQA AD/RD (%)
1	69	M	Liver segment 7/8	45/5	T2w-Nav	6	92.3/99.8
2	46	M	Liver segment 8	27.5/5	T2w-Nav	7 *	91.8/99.5
3	56	M	Liver segment 4B	27.5/5 **	T2w-Nav	5	82.1/99.3
4	74	M	Liver segment 3	30/5	T2w-Nav	8	92.4/99.8
5	71	M	Liver segment 8	40/5	T2w-Nav	10	80.9/99.5
6	85	F	Liver-2 target segment 5 & 5/6	27.5/5 35/5	T2w-Nav	3 and 4	94.8/99.9 93.2/100
7	60	M	Liver-2 targets segment 6	2 × 24/1	T2w-Nav	7 (both targets)	91.1/98.3
8	75	M	Liver-4 targets segment 5	3 × 16/1 1 × 24/1	T2w-Nav	10 (3 targets) 7 (1 target)	82.2/99.8
9	78	F	Liver Segment 8/4	30/5	T2w-Nav	6	91.5/99.5
10	63	F	Pancreas pancreatic body	30/5	T2w-Ave	8	88.0/99.0
11	80	M	Pancreas pancreatic body/neck	30/5	T2w-Nav	6	94.1/100
12	76	M	Liver segment 7	30/5	T2w-Ave	5	88.0/95.4
13	70	M	Pancreas pancreatic head	40/5	T2w-Ave	5	92.1/96.8
14	64	F	Pancreas pancreatic head/neck	30/5	T2w-Ave	5	95.6/98.8
15	58	F	Liver segment 2/3	30/5	T2w-Nav	8	96.1/100
16	77	M	Pancreas pancreatic head	40/5	T2w-Ave	6 *	93.9/98.4

* Patient received Ativan prior to the online adaptive sessions. ** Only three fractions were delivered.

## Data Availability

The data presented in this study are available on request from the corresponding author.
